# Single-item measures in digital mental healthcare: a mini narrative review of challenges and opportunities

**DOI:** 10.3389/fdgth.2026.1761107

**Published:** 2026-04-01

**Authors:** Lauren Staples, Alana Fisher, Blake Dear, Olav Nielssen, Nickolai Titov

**Affiliations:** School of Psychological Sciences, Faculty of Medicine, Health and Human Sciences, Macquarie University, Sydney, NSW, Australia

**Keywords:** cultural differences, implementation, internet delivered therapy, measurement, mental health, outcome monitoring, person-centred care, psychometric validation

## Abstract

Single-item measures (SIMs) are increasingly used by digital mental health services for assessment, outcome monitoring, and population-level surveillance. Their simplicity offers clear advantages, including good face validity, practical efficiency, and the potential to integrate across digital platforms. However, concerns persist regarding their reliability and suitability for complex psychological constructs. This mini narrative review synthesises recent literature examining the use of SIMs in mental healthcare over the past decade. A total of 31 articles underwent full-text review, and key themes were identified. Findings suggest that SIMs demonstrate acceptable validity for narrowly defined constructs and are valuable for large-scale screening and digital integration, though systematic validation across diverse populations is essential. These results suggest that SIMs should not replace comprehensive assessments, but they can complement integrated, person-centred models of measurement in digital mental healthcare.

## Introduction

Digital mental health services (DMHSs) have transformed the way mental healthcare is delivered, offering evidence-based, accessible, and acceptable psychological services to diverse populations (1–3). DMHSs can reduce the burden on mainstream healthcare systems as scalable and cost-effective adjuncts to in-person treatment options, and they have informed government and funding strategies in Australia (4–6) and internationally (1, 7–12). This progress has been largely driven by routine collection and reporting of outcomes derived from well-validated and established multi-item scales. For example, the Kessler 10-item (K-10) measure of general psychological distress demonstrates strong psychometric properties and is routinely used to monitor prevalence and trends in large-scale health surveys (13). The 9-item Patient Health Questionnaire (PHQ-9) is a reliable and valid measure of depression in general populations (14), while the Generalised Anxiety Disorder 7-item scale (GAD-7) is a psychometrically sound tool for measuring anxiety in clinical practice and research (15).

Despite their widespread use, multi-item scales can present significant challenges in routine care. The time taken to complete them can place a significant burden on respondents, who may already be experiencing symptom-related cognitive burden, and the items themselves may be ambiguous or have limited face validity (16). The length and format of multi-item scales may be misaligned with contemporary digital user experience standards, which increasingly prioritise transparency and concise, low-effort interactions (17, 18). For clinicians and researchers, the composite scoring of multi-item measures can be difficult to calculate or interpret, and item redundancy can inflate measures of internal consistency (19, 20). In rapidly scaling digital environments, these limitations may hinder timely implementation and clinical decision-making (21).

Single-item measures (SIMs) are a pragmatic alternative, due to their brevity, ease of administration, and potential for integration across digital platforms. This mini narrative review synthesises evidence for and against the use of SIMs for psychological measurement, focusing on their utility in digital mental healthcare within the last decade. Given the brief format, the review does not aim to provide an exhaustive synthesis of all available literature but instead summarises key themes and emerging insights.

## Literature search

A literature search was conducted in the following electronic databases: PubMed, PsycInfo, and Embase. The search used the keywords “single item measure” AND “mental health.” The time frame was limited to 10 years (2015–2025), and publications were restricted to English. With duplicates removed, a total of 82 articles were identified. Titles and abstracts were screened, and editorials, reviews, or research studies that lacked direct relevance to mental health assessment or outcome measurement were excluded. This screening process produced a final set of 31 articles which underwent full-text review. The final selection of studies informed the synthesis presented in this review.

## Summary of evidence

Table 1 provides an overview of the reviewed studies. Each study was categorised to indicate whether it generally supported the use of SIMs or adopted a more neutral or cautious position. Almost two-thirds of the included publications (20/31; 65%) presented evidence that endorsed the utility of SIMs as practical and acceptable options for measurement in clinical and research settings. These studies commonly highlighted advantages such as reduced respondent burden, ease of administration, and suitability for integration into digital platforms. The remaining studies (11/31; 35%) acknowledged some of the advantages of SIMs, but also highlighted significant limitations, particularly regarding their reliability, validity, and generalisability across populations or diagnostic groups. From this body of literature, three key themes emerged as important considerations when examining the strengths and limitations of SIMs in digital mental healthcare.

**Table 1:** Summary of studies evaluating single-item measures (SIMs) in mental health.

First author and year	SIM	Study population(s)	Key findings	Support for SIM
Ammerman et al. (22)	Multiple SIMs for suicidal ideation, planning, and attempts. Response time frames included past month, past year, and over the lifetime.	Adults recruited from an online community (*n* = 613).	Examined the impact of wording and response time frames on SIM consistency. Minor wording changes significantly altered endorsement rates and therefore prevalence estimates. Cautioned against the use of SIMs for assessing complex or sensitive constructs.	Cautious
Assari (23)	“How would you rate your overall physical health?” Responses: excellent, very good, good, fair, or poor.	US adults compared across 10 ethnic groups (*n* = 18,237).	Examined the relationship between psychiatric disorders and an SIM for self-rated physical health. Found that the SIM did not exclusively measure physical health but was biased by mental health status in some ethnic groups. Highlighted the need to consider cultural and ethnic differences when creating and using SIMs.	Cautious
Botha et al. (24)	“During the past week, about how often did you feel depressed or anxious?” 1—none of the time; 2—a little of the time; 3—some of the time; 4—most of the time; 5—all of the time.	Australian adults accessing an online survey during the COVID-19 pandemic (*n* = 1,158).	Examined the psychometric properties of an SIM for general distress compared to the multi-item Kessler Psychological Distress Scale (K-6). SIM showed strong correlation with the K-6 and high predictive value. Concluded that SIMs which are validated against established scales are useful for monitoring trends and are a practical option when respondent burden or cost are concerns.	Supportive
Campbell et al. (25)	Seven SIMs related to social-emotional problems. Various response options.	Australian First Nations children aged 3–16 years (*n* = 84).	Compared SIMs related to social and emotional wellbeing against the Behaviour Assessment System for Children-3rd edition (BASC-3). SIMs generally showed good convergent validity and sensitivity, although sensitivity for anxiety was low. Demonstrated the cultural responsiveness of SIMs in routine monitoring, but noted that wording of the item related to anxiety may not be culturally appropriate or relevant.	Cautious
Cloos et al. (26)	Multiple SIMs for positive and negative affect, with various response options.	Dutch-speaking participants with a mean age of 22 years (*n* = 153).	Developed and validated a new questionnaire that included both single- and multi-item measures of affect. SIMs showed strong psychometric properties. Concluded that SIMs are valid and efficient tools that can be used in longitudinal studies to assess affective states relevant to mental health.	Supportive
Cullati et al. (27)	Three SIMS for self-rated health, with four coding schemes.	Respondents of the Swiss Health Survey, aged 15 years and older (*n* = 58,023).	Examined construct validity equivalence across three variations of an SIM for self-rated health. Differences in wording and response coding significantly affect construct validity, particularly for the mental health dimension. The best-performing SIM used the following wording: “How is your health status in general?” Linear coding of responses as continuous variables maximised construct validity equivalence across the SIMs.	Cautious
Edwards et al. (28)	Two SIMs assessing the impact of pain. One used a 5-point response scale and one used an 11-point scale.	Treatment-seeking adults with mixed-aetiology chronic pain (*n* = 633).	Examined psychometric properties of two SIMs assessing the impact of pain on functioning. Both SIMs demonstrated strong concurrent, discriminant, and predictive validity, and were predictive of depression. Supported the utility of low-burden SIMs in clinical and research settings.	Supportive
Fisher et al. (29)	Thirty-seven SIMs for various constructs, including a newly developed SIM for perceived depression: “In the past month, how often have you felt depressed?”	Online survey respondents (*n* = 1,634), and subject matter experts (*n* = 17).	Examined the psychometric properties of multiple SIMs. The SIM for depression showed moderate reliability and convergent validity, and moderate short-term test–retest reliability. Concluded that SIMs have some practical advantages, but multi-item measures are preferable from a psychometric perspective.	Cautious
Fung (30)	"How would you rate your overall mental health?” Rated from 1 (poor) to 5 (excellent).	Two convenience samples of adults from Hong Kong and/or Taiwan (*n* = 205; *n* = 377), and a random sample of psychiatric inpatients from Taiwan (*n* = 100).	Examined the reliability of an SIM for mental health. The SIM showed moderate to good test–retest reliability and correlated significantly with self-esteem, depression, and other mental health symptoms. Supported the utility of the SIM for specific cultural groups.	Supportive
Hughes et al. (31)	Three SIMs related to sleep difficulties. Rated on a 5-point Likert scale.	US military veterans (*n* = 1,118).	Evaluated the utility of SIMs for sleep difficulties and the relationship between sleep and mental health. SIMs showed moderate sensitivity and acceptable rates of false positives and false negatives. Concluded that SIMs can flag cases for further evaluation, but should not replace full diagnostic evaluations.	Cautious
Jha et al. (32)	SIM measuring of non-work-related activity impairment on a scale of 0–10.	US adults with major depressive disorder, aged 18–75 years (*n* = 2,697).	Evaluated the prognostic utility of an SIM of daily functioning. Found that it measured functional change independent of symptom changes, in response to antidepressant treatment. Concluded that the inclusion of SIMs in clinical settings can help predict treatment outcomes.	Supportive
Jovanović (33)	″All things considered, how satisfied are you with your life as a whole?," with a 10-point scale from 1 (not at all satisfied) to 10 (completely satisfied).	Three samples of Serbian adolescents (*n* = 481, *n* = 283, and *n* = 220).	SIM for life satisfaction compared with multi-item scale. SIM showed good psychometric properties, and moderate correlations with indicators of mental health and wellbeing. Demonstrated the utility of an SIM for life satisfaction in adolescents.	Supportive
Keding et al. (34)	"Over the last week how depressed have you felt on average? Please reply with a score between 1 and 9; where 1 is ‘not at all’ and 9 is ‘extremely.’”	UK adults with symptoms of depression (*n* = 373).	SIM for depression delivered via SMS, validated against the 9-item Patient Health Questionnaire (PHQ-9). SIM demonstrated good psychometric properties. Concluded that it can be used to monitor depression, but requires further validation in diverse populations and contexts.	Supportive
Kim and Abraham (35)	"I felt depressed," rated from 0 to 4. A second SIM asked respondents to rate their depression from 0 (none) to 10 (unbearably severe).	Young adults aged 19–37 years, attending nursing colleges in Korea (*n* = 458).	Compared reliability and validity of SIM of depression to multi-item measures. SIMs showed weak psychometric properties. Concluded that they may not adequately capture the complexity of depression, and highlighted the need to consider cross-cultural differences.	Cautious
Lin et al. (36)	“How would you rate your present health status?” “In general, how would you rate your current state of happiness?” Both rated on a scale of 0–100.	Taiwanese adults aged 65 years or older (*n* = 3,982).	Compared SIMs for assessing physical and mental health against multi-item measures in older adults. SIMs showed good validity, but age and gender differences should be considered when evaluating this population.	Supportive
Macias et al. (37)	"In general, would you say your health is: excellent, very good, good, fair, or poor?"	Psychiatric outpatients with co-occurring chronic physical conditions (*n* = 177).	SIM for global health was evaluated relative to the SF-36 General Health Scale. SIM showed strong discriminant validity and was sensitive to change. Concluded that SIMs can be as valid and reliable as multi-item scales for longitudinal research.	Supportive
Maguire et al. (38)	“How would you rate your mental health in general?” “How would you rate your health in general?” 1—poor; 2—fair; 3—good; 4—very good; 5—excellent.	Australian adults diagnosed with schizophrenia (*n* = 71).	Examined the correlates of an SIM for mental health in people with schizophrenia. The SIM was associated with higher psychological distress, negative illness perceptions, and increased anxiety and depression. Concluded that SIMs can be used as brief screening tools to identify vulnerabilities in a specific cohort of people with a high level of health-related needs.	Supportive
McClure et al. (39)	Multiple SIMS for suicidal ideation, planning, and attempts. Yes/no response across various time frames.	US adults compared across racial and ethnic groups (*n* = 1,624).	Examined the measurement invariance of SIMs related to suicidal thoughts and behaviours. SIMs were generally reliable and comparable across groups within study without bias. However, wording affected predictive validity, limiting comparability across studies. Highlighted the need to test validity and precision across culturally diverse groups.	Cautious
Min et al. (40)	"Have you felt sad or depressed for more than two weeks continuously in the past year?"	Older Korean adults aged between 60 and 89 years (*n* = 800).	SIM for depression evaluated against multi-item measures. SIM significantly underestimated depression prevalence and exhibited low sensitivity. Concluded that it was not a reliable way of identifying depression in older adults, particularly those with lower educational levels.	Cautious
Mund et al. (41)	Multiple SIMs for loneliness, with various response options.	Three samples of US adults (*n* = 697; *n* = 1,216 and *n* = 411).	Psychometric properties of SIMs for loneliness were assessed. SIMs showed good reliability and validity, and an association between loneliness and depression. Concluded that SIMs can be used effectively in research contexts where time or financial constraints make the use of multi-item scales impractical.	Supportive
Nair et al. (42)	SIMs for general health and mental health. Rated from “poor” (1) to “excellent” (5).	Patients accessing early psychosis services in India (*n* = 159) and Canada (*n* = 102).	Psychometric properties of SIMs for general health and mental health. Results were mixed, and highlighted the need to consider cultural, socioeconomic, and other contextual factors when using SIMs in clinical settings.	Cautious
Richmond et al. (43)	“Over the last week how depressed have you felt on average? Please reply with a score between 1 and 9; where 1 is ‘not at all’ and 9 is ‘extremely’."	Patients with depression recruited from primary care (*n* = 527).	Developed and assessed an SIM for depression delivered via automated text messaging. Feasibility, acceptability, and validity were high, and the SIM appeared more responsive to treatment change than the PHQ-9. Highlighted the utility of the SIM as a reliable outcome measure.	Supportive
Song et al. (44)	Multiple SIMs with various response options.	Secondary analysis of individuals with a primary diagnosis of anxiety and/or depression (*n* = 45).	Investigated the psychometric properties of multiple single- and multi-item measures related to mental health. The SIMs showed adequate concurrent and predictive validity comparable to multi-item measures. Highlighted the utility of SIMs in reducing participant burden, particularly in longitudinal research.	Supportive
Stubb et al. (45)	"In general, would you say that your mental health is excellent, very good, good, fair, poor or very poor?"	Australian healthcare workers (*n* = 385).	Examined associations between an SIM for mental health and Kessler 10-item measure of psychological distress (K-10). Showed that the SIM was an efficient screening tool, highlighting its potential for monitoring mental health.	Supportive
Turon et al. (46)	"Over the past week have you: a) Felt anxious? (Yes/No); and b) Felt depressed? (Yes/No)"	Australian outpatients with cancer (*n* = 2,811).	Evaluated SIM for anxiety and depression against the 14-item Hospital Anxiety and Depression Scale (HADS). SIM showed moderate sensitivity and specificity, moderate positive predictive values, and high negative predictive values. Highlighted the efficiency of the SIM as tool for screening out individuals from further psychological assessment, in a clinical setting.	Supportive
Verlenden et al. (47)	"During the past 12 months, did you ever feel so sad or hopeless almost every day for 2 weeks or more in a row that you stopped doing some usual activities? (Yes/No)”	US adolescents aged 15–19 years (*n* = 737).	SIM for sadness/hopelessness was significantly associated with other indicators of poor mental health, including moderate–severe depression, mental distress, and functional limitations. Highlighted the utility of the SIM for population-level monitoring, to identify unmet health-related needs and inform public health decisions.	Supportive
Verster et al. (48)	Multiple SIMs with various Likert scale response options.	Dutch adults, aged 18–30 years, recruited online (*n* = 2,489).	Compared SIMs and multi-item scales and subscales. SIMs for depression and mood showed adequate agreement with corresponding multi-item subscales, but agreement diminished for more complex constructs. Concluded that SIMs are a practical tool for evaluating some, but not all, health-related constructs.	Cautious
Williams and Smith (49)	"On a scale of one to ten, how anxious would you say you are in general? (e.g., feeling tense or ‘wound up’, unable to relax, feelings of worry or panic)?” “On a scale of one to ten, how depressed would you say you are in general? (e.g., feeling ‘down’, no longer looking forward to things or enjoying things that you used to)?”	UK university staff members aged 20–64 years (*n* = 120).	Evaluated SIMs for anxiety and depression against the HADS. SIMs showed good diagnostic validity, and concluded that they are suitable for rapidly identifying clinical cases of anxiety and depression.	Supportive
Wong et al. (50)	“Please indicate the level of stress which you consider you have experienced in the past one month on a scale of 0–10” (0 = not at all and 10 = extremely).	Two samples of young people in Hong Kong (*n* = 1,445 and *n* = 258).	Evaluated the psychometric properties of an SIM for subjective stress. The SIM demonstrated good content and face validity, and was strongly correlated with a range of mental health outcomes. Findings support the value of SIMs as early screening tools.	Supportive
Yalçın et al. (51)	"How would you rate your overall sense of well-being and mental health?” “How would you describe your child's mental health?” Both use the present as the time frame and a 5-point Likert scale.	Child–mother pairs (*n* = 411) recruited from Turkish tertiary care hospitals.	Examined the psychometric properties of SIMs assessing parent and child emotional wellbeing. Both SIMs showed good psychometric properties. Concluded SIMs can be reliable and efficient tools for mental health screening in clinical settings, offering a practical alternative to more complex assessments.	Supportive
Young et al. (52)	“Over the last week, how much have you been bothered by feeling sad, down or uninterested in life?” “Over the last week, how much have you been bothered by feeling anxious or nervous?”	Cardiac inpatients with a mean age of 66 years (*n* = 105).	Compared SIMs for anxiety and depression with the HADS. Both SIMs were highly correlated with the corresponding subscale from the HADS, and showed robust receiver–operator characteristic curves. Concluded that the SIMs are convenient and free tools that accurately identify patients experiencing anxiety and depression.	Supportive

## Key themes

### SIMs have acceptable validity and reliability when measuring clearly defined constructs

Evidence suggests that SIMs demonstrate acceptable psychometric properties when measuring narrow, clearly defined, and unidimensional constructs (48). Several studies reported strong correlations between SIMs and multi-item scales for depression (49, 52), anxiety (49, 52), and psychological distress (24, 49, 53). However, even minor changes in wording significantly influenced construct validity when measuring non-specific dimensions of general health (27) or sensitive mental health constructs such as suicidal thoughts and behaviours (22). These results underscore the need for systematic validation protocols (29, 34).

### SIMs demonstrate practical utility for large-scale screening and digital integration

Quickly identifying unmet need, particularly in vulnerable cohorts, is a key public health activity and is critical for informing government and policy decision-making (32, 38, 45, 47, 54). SIMs offer practical advantages for population-level monitoring and rapid assessment, including reduced respondent burden and increased cost-effectiveness (28, 34, 37, 43, 44, 46, 51, 55). However, several studies demonstrated that sociodemographic factors, such as age, gender, and education, affected the performance of SIMs (36, 40, 41), suggesting that contextual factors need to be considered when developing and implementing SIMs (31, 33, 36, 40, 42).

### Cross-cultural generalisability of SIMs

The brevity of SIMs can facilitate translation and interpretation, making large-scale international, cross-cultural studies feasible. However, this does not negate the need to validate them across languages and cultural contexts. Several studies, presented in Table 1, reported that SIMs perform well across a range of diverse populations (26, 30, 50). However, others found that SIMs did not adequately capture the nuances associated with mental health constructs in heterogeneous ethnic and cultural groups (23, 25, 35, 39). These results highlight the importance of measurement invariance testing to ensure conceptual equivalence when translating SIMs (39).

## Future directions

### Person-centred care

Person-centred healthcare recognises the preferences, needs, and values of patients and consumers, and is integral to the development, implementation, and evaluation of health services (56). The brevity and accessibility of SIMs position them as effective tools for promoting user engagement and shared decision-making (42). Designing SIMs to support person-centred approaches requires attention to health and digital literacy, cultural context, and codevelopment with consumers, particularly those belonging to vulnerable or diverse populations (57). Future research that prioritises consumer engagement will strengthen the evidence base, allowing policymakers and funders to make informed decisions about scalable, person-centred care.

In addition, DMHSs are rapidly evolving in conjunction with other emerging and existing technologies, including smartphones, artificial intelligence, and virtual reality. There is significant potential for the development of models that integrate SIMs with these technologies (58). Future research should focus on implementation evaluations and standardisation frameworks to ensure interoperability across platforms to support data aggregation and large-scale analytics.

### Stakeholder alignment

Stakeholder alignment remains an important consideration for future development of SIMs. Clinicians, researchers, and consumers may hold different expectations about what SIMs can and should capture. For clinicians, SIMs may function as triage tools or performance metrics; for researchers, they may provide efficient instruments for assessment and treatment outcomes; and for consumers, they may offer an accessible way to express health and wellbeing without completing lengthy questionnaires. Aligning these expectations is critical to ensuring that SIMs are used appropriately and interpreted safely within clinical pathways. Clear communication about the role and limitations of SIMs—including how scores are used and what actions they trigger—can help mitigate risks associated with over-interpretation or over-reliance on a single indicator.

## Limitations

This review provides a brief snapshot of peer-reviewed publications relevant to the use of SIMs in DMHSs. As a mini review of literature published in the last decade, the focus is on a narrow set of studies rather than an exhaustive summary. We also acknowledge that the methodological quality or risk of bias in the included articles has not been assessed. Given these constraints, the conclusions drawn here should be interpreted as indicative rather than definitive. A systematic review incorporating broader search strategies and structured methodologies would help clarify whether variations in SIM performance reflect true construct differences, population-specific factors, or inconsistencies in operationalisation, phrasing, or response format.

## Conclusion

Despite these limitations, this synthesis of findings suggests that SIMs may be a pragmatic option for monitoring symptoms and outcomes in digital mental health, offering scalability, accessibility, and reduced burden for respondents and providers. Their brevity, ease of administration, and compatibility with digital workflows make them well suited for high-frequency monitoring, large-scale screening, and stepped-care triage. Moreover, the reduced burden on respondents can improve completion rates, minimise survey fatigue, and support more continuous engagement—considerations which are often particularly relevant for populations experiencing psychological distress, cognitive load, or health-related functional impairments. However, further research is necessary to ensure generalisability, psychometric validation, and stakeholder alignment. Importantly, SIMs should not be considered replacements for multi-item scales. Instead, SIMs may serve as practical tools to enable data-driven decision-making at scale and support hybrid models of measurement, balancing efficiency and depth. As part of a digital mental health framework, SIMs may function as tools for triage, screening, or monitoring, while multi-item scales continue to provide the granularity needed for diagnosis, treatment planning, and rigorous outcome evaluation.

In conclusion, SIMs hold promise as low-burden, scalable components of DMHSs. To realise this potential, further research is needed to assess their reliability across populations, ensure cross-cultural validity, evaluate their role within stepped-care decision frameworks, and examine their impact on clinical outcomes when used in routine practice. With careful implementation, stakeholder engagement, and ongoing validation, SIMs may meaningfully contribute to more responsive, person-centred, and data-informed models of mental healthcare.
